# Regular intake of white kidney beans extract (*Phaseolus vulgaris* L.) induces weight loss compared to placebo in obese human subjects

**DOI:** 10.1002/fsn3.1299

**Published:** 2020-02-05

**Authors:** Shenli Wang, Lishui Chen, Haiying Yang, Jinghan Gu, Jing Wang, Fazheng Ren

**Affiliations:** ^1^ Beijing Advanced Innovation Center for Food Nutrition and Human Health College of Food Science and Nutrition Engineering China Agricultural University Beijing China; ^2^ Brand Food R&D Center Nutrition & Health Research Institute (COFCO‐NHRI) Beijing China; ^3^ Beijing Engineering Laboratory of Geriatric Nutrition & Foods Beijing China; ^4^ Beijing Key Laboratory of Nutrition & Health and Food Safety Beijing China; ^5^ Beijing Advanced Innovation Center for Food Nutrition and Human Health School of Food and Health Beijing Technology & Business University Beijing China

**Keywords:** obesity, *Phaseolus vulgaris* L., weight loss, α‐Amylase inhibitor

## Abstract

**Scope:**

*Phaseolus vulgaris* L. is rich in alpha‐amylase inhibitor and has been used for reducing glycemia and calories absorption through preventing or delaying the digestion of complex carbohydrate. A randomized, double‐blinded, placebo‐controlled study was conducted on obese volunteers to evaluate the degree of significate weight loss by regular intake *Phaseolus vulgaris* cultivated from Southwestern region of China.

**Method:**

The volunteers were divided into two groups, homogeneous for age, gender, and body weight. *Phaseolus vulgaris* extract or placebo was given 2,400 mg per day before each daily meal for 35 consecutive days. Each subject's body weight, fat mass, body mass index, blood biochemical parameters, skinfold fat thickness, and waist/hip circumferences were monitored and analyzed.

**Result and conclusion:**

As a result, the average amount of weight lost by the *Phaseolus vulgaris* extract group was 2.24 kg (average of 0.448 kg per week), compared with a 0.29 kg weight loss (average of 0.058 kg per week) in placebo group after 35 days. The differences between groups were significant (*p* < .01). The body mass index decreased by an average of 0.79, and the body fat decreased by 1.53% on average compared to baseline (*p* < .05). The thickness of subcutaneous fat was significantly reduced at the four measurement points, and the decrease of waist circumference and hip circumference was significant as well. No adverse or side effects were observed during the trial period. The results indicate that *Phaseolus vulgaris* extract can significantly induce weight loss in a short time period.

## Graphical abstract

A *Phaseolus vulgaris* extract produced from Southwestern region of China reduces body weight gain in obese subjects. There is no serious side effects report following consumption of *Phaseolus vulgaris* extract. *Phaseolus vulgaris* extract has the potential to induce weight loss caused by carbohydrates through its alpha‐amylase inhibiting activity.

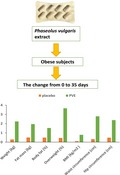

## 1 INTRODUCTION

The World Health Organization (WHO) estimated that more than 1.9 billion adults (39% of men and 40% of women) aged 18 years and older were overweight in 2016 and of these over 650 million adults (11% of men and 15% of women) were obese. The worldwide prevalence of obesity nearly tripled between 1975 and 2016. Globally, there are more people who are obese than underweight—this occurs in every region except parts of sub‐Saharan Africa and Asia (WHO: Obesity & Overweight, 2018). Overweight and obesity are related to more deaths worldwide than underweight. The overweight and obesity states increase the risk of hypertension, type 2 diabetes, arthritis, sleep apnea, dyslipidemia, cardiovascular diseases, various types of cancer, and premature death (van Dam & Seidell, 2007; Willett, Dietz, & Colditz, 1999). Therefore, the importance of prevention and treatment of obesity is widely acknowledged. Due to the obesity is the imbalance of energy intake and expenditure, effects of diet on body energy intake are one of obvious targets for intervention. Food science researchers have investigated many plant‐derived foods consumption for decreasing energy intake on both animal experiments and human studies, trying to offer a promising therapy to ameliorate obesity and its complication.

The white kidney beans are one species of *Phaseolus vulgaris* L., also known as common beans, is originated from South American countries, such as Mexico and Argentina. Around 23 million tons were produced in the world in 2012, and the largest consuming region is South America (Heredia‐Rodríguez, de la Garza‐Juárez, Vazquez‐Rodriguez, 2017). It has been cultivated and domesticated to adapt to the cold and humid plateau. Nowadays, the white kidney beans are widely planted in many regions of China, the provinces with large cultivate area is Yunan, Guizhou, and Sichuan. In recent decades, the white kidney beans have been observed as a nutraceutical because it contains a bulk of bioactive compounds, such as polyphenols, resistant starch, oligosaccharides, and bioactive peptides nutrients (Heredia‐Rodríguez et al., 2017). *Phaseolus vulgaris* beans are rich in proteins (22%–27% of seed weight) and carbohydrates (39%–47% of seed weight), thus, making them valuable food ingredient for over half a billion people (He et al., 2015). Thus, 100 g of dry common beans serve in human provides about 9–25 g of protein, which is almost 20% of the recommended daily consumption for a normal adult. Consequently, the protein present in the beans meets the minimal need of human requirements endorsed by the WHO and Food and Agriculture Organization.

At present, there are a large number of evidences demonstrated that *phaseolus vulgaris* extract (PVE) have potential effects on human health and possess antioxidant, anticarcinogenic, anti‐inflammation, antiobesity, antidiabetes, and cardioprotective properties (Ganesan & Xu, 2017; Garcia‐Mora et al., 2015; Kumar & Baojun, 2017; Obiro, Zhang, & Jiang, 2008; Oseguera‐Toledo, de Mejia, Dia, & Amaya‐Llano, 2011). A number of laboratory animal studies indicated that acute or chronic administration of the derivatives, extracts, and ingredients of PVE significantly reduces appetite, food intake, carbohydrate absorption and metabolism, lipid accumulation, body weight gain, glycemia, and glucose absorption and modulation of gut microbiota in lean and obese animals (Fantini et al., 2009; Maccioni et al., 2010; Pusztai et al., 1998; Song et al., 2016). It also has been shown that administration of the amylase inhibitor purified from PVE reduced blood glucose levels, body weight gain, and postprandial hyperglycemia in both the nondiabetic and diabetic animals (Tormo, Gil‐Exojo, Romero, & Campillo, 2004; Tormo, Gil‐Exojo, Romero de Tejada, & Campillo, 2006). A series of clinical trials also support that PVE may constitute a potentially safe and effective remedy for the treatment of obesity and associated metabolic syndromes. Subjects receiving PVE had significant reduction of body weight, body mass index (BMI), fat mass, adipose tissue thickness, and waist/hip/thigh circumferences while maintaining lean body mass compared to subjects receiving placebo (Celleno, Tolaini, D'Amore, Perricone, & Preuss, 2007; Udani & Singh, 2007; Wu, Xu, Shen, Perricone, & Preuss, 2010). Regular consumption of PVE is also beneficial to prevention of diabetes and inversely associated with the risk type 2 diabetes (Villegas et al., 2008). Studies show that purified PVE supplementation reduces postprandial glucose, insulin, C‐peptide excursions, appetite, and suppressed ghrelin secretion in healthy human subjects (Spadafranca et al., 2013).

The PVE is rich in phaseolin, classical α‐amylase inhibitors (α‐AI), and known as "starch blocker." The α‐AI is a natural bioactive component belonging to the class of glycoside hydrolase and has antiamylase activity in humans (Moreno, Altabella, & Chrispeels, 1990). The α‐AI interferes with the breakdown of complex carbohydrate leading to a reduced digestibility or prolonged digestion. Therefore, energy derived from the carbohydrate and the rate of body absorption of the energy in form of glucose are reduced (Celleno et al., 2007). α‐AI can inhibit the activity of saliva and pancreatic amylase in the gastrointestinal tract, impede or delay the hydrolysis, and digestion of the main carbohydrates in food and reduce the decomposition and absorption of starch sugars in foods, thereby reducing body weight, blood sugar, and blood lipids (Song et al., 2016). The role of inhibiting the increase in blood glucose concentration is conducive to diet treatment of diabetic patients. For obese patients, sugar‐to‐fat conversion was reduced, intestinal emptying was delayed, and fat consumption was reduced leading to reduce body weight. α‐AI could be used to prevent and treat obesity, hyperlipidemia, arteriosclerosis, hyperlipidemia, and diabetes. A study of two kinds of *Phaseolus vulgaris* (pinto Durango and black 8,025) indicate that *Phaseolus vulgaris* polypeptide (molecular weight < 1 kDa) increased insulin secretion, reduced DPP‐IV protein and RAGE receptor expression, improved dyslipidemia, and passed Akt signaling pathways increase glucose uptake (67%) and improve insulin resistance (Toledo, Mejia, Sivaguru, & Amaya‐Llano, 2016). McCrory et al. show that regular intake of *Phaseolus vulgaris* containing soluble fibers and resistant starch decreased the body's glycemic index, reduced low‐density lipoprotein (LDL), increased high‐density lipoprotein (HDL) levels, and positively influences risk factors for metabolic syndrome, thereby reducing the risk of cardiovascular disease (CVD), obesity, and diabetes (Mccrory, Hamaker, Lovejoy, & Eichelsdoerfer, 2010). Therefore, PVE have outstanding advantages in slowing the breakdown of complex carbohydrates, reducing the absorbed by the gut and influencing the glucose‐insulin system, thus promoted prospects in the treatment of diseases such as obesity and diabetes.

Although some clinic studies demonstrated that the PVE exerted beneficial effects in weight reduction, other studies showed there is no significant effect in body fat or body measurements (waist/hip circumferences) compared to placebo. The effects of PVE on weight loss indicators are still contradictory in clinical studies. A brand PVE product was identified for weight loss and a decrease in triglycerides, but statistical significance was not reached in body fat percentage, energy level, waist/hip circumferences, and total cholesterol (Udani, Hardy, & Madsen, 2004). The potential dietary therapy of PVE as a functional food ingredient for treatment of obesity needs further studies with large number of subjects to demonstrate effectiveness. The objective of the present study was to evaluate the influence of administrated PVE in capsule form before daily meals on weight loss and side effects or adverse events in obese subjects. Based on our knowledge, we have firstly investigated the effects of PVE cultivated from Southwestern region of China on the potential value as dietary functional ingredient for treatment of obesity.

## 2 METHODS

### 2.1 Subjects and study design

A total of 120 obese volunteers (60 women and 60 men; aged between 18 and 65 years) were enrolled into this study. Inclusion criteria were as follows: simple obesity, BMI ≥ 30, or total fat percentage reaching: men > 25% and women > 30%. Exclusion criteria were as follows: participants had history in severe diseases such as heart, liver, kidney, digestive system, hematopoietic systems, and psychotic system and any other disease that could influence the study results; the subjects who receive pharmacological treatment or used any other product may affect the judgment of the results in the short term; Subjects who are not consumed test products as prescribed. The present study was conducted in accordance with The Code of Ethics of the World Medical Association (Declaration of Helsinki) for experiments involving human subjects and approved by the Clinic Research Ethics Committee of Guangxi Zhuang Autonomous Region for Disease Prevention and Control.

Volunteers were randomly divided into two groups according to body weight and body fat weight: sixty to control group start the trial with the placebo and sixty to intervention group with PVE. At the end of the trial, the valid number for each group was not less than 50 subjects. In order to avoid any bias due to gender, the number of men and women were balanced. The PVE was administered in the form of capsule and provided by the Yunnan Tianbaohua Biological Resources Development Co., Ltd, 400 mg/capsule, stored in a cool dry environment. The intervention group was advised to take PVE capsules according to recommended amount for human: 2 capsules (400 mg per capsule) each time before 3 daily meals, for a total of 2,400 mg per day and 35 consecutive days. The main constituent of PVE was protein, up to 75.4 ± 1.2 gram per 100 gram of the extract (g/100g). Carbohydrates were also detected with content of 14.5 ± 0.6 g/100g. And the detection of fat showed that it was 2.8 ± 0.2 g/100g. Besides, the product contains less than 150 hemagglutinating units/g, phytohemagglutinin, and α‐amylase inhibitor with 1,360 ± 30.2 U/g inhibiting activity. The placebo containing maltodextrin (1632 kJ/100g) was used in the control group. The method of administration was the same as that of the intervention group. Placebo's appearance, texture, taste, smell, and packaging are consistent with the extract. Blood samples, blood pressure, heart rate, and anthropometric measurements were taken at baseline and at the end of intervention.

### 2.2 Dietary control and exercise observation

Subjects were assigned to maintain the same dietary habits along the study period. Before the trial and in the last week of study period, volunteers were asked to complete a three‐day detailed food intake report, specifying the ingredients and amounts of consumed food and serving weights. Volunteers were asked to fill out a questionnaire before starting the study in order to know their occupation and leisure time activities, and consequently the physical activity involved, and keep consistent with the daily exercise conditions during the study period.

### 2.3 Blood biochemical biomarker analyses

Blood samples were collected after 8–10 hr of overnight fasting at baseline and at the end of the PVE intervention stages. Serum (without anticoagulant) and plasma (EDTA‐coated tubes) were separated by centrifugation and frozen at −80C until analysis. Serum albumin, total protein, aspartate aminotransferase (AST), alanine aminotransferase (ALT), serum glucose, blood urea nitrogen (BUN), uric acid, and creatinine levels were analyzed according to standardized spectrophotometric techniques.

### 2.4 Athletic endurance analysis

The maximum oxygen consumption (L/min) for each subject was estimated by Astrand‐Ryhming nomogram, using the cycle ergometer as the sole exercise mode and following the recommended submaximal test protocol (Cink & Thomas, 1981). The subjects stepped on the exercise bike (Monark 828E Test Ergomedic) with the same work load at baseline and at the end of each intervention. The preset exercise power was 100 watts for both men and women and records the heart rate at the end of 5th minute exercise.

### 2.5 Anthropometric analysis

The BMI was calculated according to the formula weight (kg) per height (m)^2^. Standard weight and overweight were calculated by using the following formula: Adult standard weight (kg) = (height (cm) – 100) * 0.9, Overweight (%) = (weight (kg) – standard weight (kg))*100/ standard weight (kg). Total body fat (kg) and body fat percentage were determined by using a body fat analyzer (Tanita Tbf‐410A body composition analyzer, Japan). Subcutaneous fat thickness was measured by two trained examiners using a Lange caliper (Beta Technology Inc.), and three measurements were taken from the following sites on the right side of the body. (a) Triceps, halfway between the acromion process and the olecranon process; (b) subscapular, about 20 mm below the tip of the scapula, at an angle of 45 to the lateral side of the body; (c) abdomen, 3 cm next to the right umbilicus; (d) suprailiac, about 20 mm above the iliac crest, in the axillary line.

### 2.6 Statistical analysis

SPSS version 22.0 program was used for the statistical analysis of data. Homogeneity of variances was checked by the test of Levene. Differences between the Placebo and PVE group at each time point were analyzed using Student's *t* test. The level of significance was *p* < .05.

## 3 RESULTS

One hundred and twenty cases of simple obesity were divided into two groups to perform weight‐loss intervention. At the end of the trial, 6 cases were withdrawn, lost to follow‐up, and failed to take samples as required. The rate of withdrawal was 6.0%. Finally, the effective statistics groups were as follows: 58 volunteers (28 men and 30 women) receiving placebo as control group; 56 volunteers (29 men and 27 women) receiving PVE as active group. There was no significant difference in height and weight between the two groups at baseline (*p* > .05), as is shown in Table 1. During the trial, the two groups of subjects had normal psychiatric status, and no abnormalities were observed in diet, sleep, and urine.

**Table 1:** Baseline characteristics of obese volunteers (Data are shown as means ± standard deviation, PVE: *phaseolus vulgaris* extract)

	Placebo (*n* = 58)	PVE (*n* = 56)	*p* value (between groups)
Age (years)	42.9 ± 8.0	42.4 ± 8.5	>.05
Height (cm)	170.4 ± 5.6	169.1 ± 5.4	>.05
Weight (kg)	83.81 ± 4.72	82.87 ± 4.75	>.05
Body fat (kg)	26.18 ± 2.43	26.61 ± 2.13	>.05

### 3.1 Dietary recording

A three‐day dietary survey before and after the start of the intervention was performed on obese subjects. The daily diet and energy intake showed no statistical differences due to the placebo and PVE treatments, as shown in Table 2.

**Table 2:** Dietary recording and energy intake before and after trial in two groups of subjects. (Data are shown as means ± standard deviation)

Diet	Control (*n* = 58)			Active (*n* = 56)		
	Baseline	Placebo	*p* Value	Baseline	PVE	*p* Value
Rice and noodle (g/day)	315.3 ± 15.4	313.3 ± 14.0	>.05	316.7 ± 16.5	308.8 ± 15.8	>.05
Livestock, poultry, and internal organs (g/day)	122.8 ± 8.5	120.5 ± 9.7	>.05	125.1 ± 12.0	120.6 ± 9.4	>.05
Egg and fish shrimp (g/day)	110.9 ± 15.7	112.2 ± 12.4	>.05	111.9 ± 12.7	109.1 ± 10.8	>.05
Fruits (g/day)	323.3 ± 16.4	321.5 ± 16.0	>.05	324.7 ± 22.0	317.2 ± 21.8	>.05
Energy (Kcal/day)	2,372 ± 192.5	2,356 ± 177.2	>.05	2,388 ± 206.8	2,340 ± 181.5	>.05

### 3.2 Effects on heart rate and blood pressure

During the initial and intervention period of the trial, the majority of subjects’ heart rate and blood pressure were within the range of normal values (Table 3). Some subjects were mildly hypertensive (diastolic blood pressure between 90 mmHg and 100 mmHg) due to obesity, but all of them are in a stable and under controllable state. No abnormal fluctuations have been observed during the intervention period.

**Table 3:** Effects of intervention with placebo and PVE on heart rate and blood pressure test in obese subjects (Data are shown as means ± standard deviation)

	Placebo (*n* = 58)			PVE (*n* = 56)		
	Baseline	35 Days	*p* Value	Baseline	35 Days	*p* Value
Heart rate (times/min)	78.3 ± 6.0	78.3 ± 6.1	>.05	80.5 ± 5.6	80.0 ± 5.6	>.05
Systolic blood pressure (mmHg)	126.4 ± 5.4	126.6 ± 5.7	>.05	125.1 ± 12.0	120.6 ± 9.4	>.05
Diastolic blood pressure (mmHg)	88.3 ± 5.5	88.3 ± 5.8	>.05	127.7 ± 6.0	127.0 ± 6.3	>.05

### 3.3 Effects on urine, routine, and hematology parameters

The side effects or adverse effects of intake extracts and placebo were also evaluated during the observation period (data are not shown). Before and after the intervention meal, no abnormalities were found in the color, traits, and microscopic examination of feces in the two groups of subjects. There were no abnormalities in urine pH, transparency, color, and urine sedimentation at baseline and at the end of trial between the two groups. The urine ketone was negative.

Before and after the trial, there was no statistically significant change in the red blood cell, white blood cell, platelet count, and hemoglobin in both active and placebo groups. There was no significant difference in intergroup and intragroup at the beginning and the end of intervention (*p* > .05) (Table 4). Hematology results were considered to be nonadverse to subjects when oral administration of PVE.

**Table 4:** Effects of intervention with placebo and PVE on hematology parameters in obese subjects (Data are shown as means ± standard deviation)

	Normal value	Placebo (*n* = 58)			PVE (*n* = 56)		
		Baseline	35 Days	*p* Value	Baseline	35 Days	*p* Value
Red blood cell (*10^12^/L)	3.5–5.0	4.89 ± 0.37	4.75 ± 0.75	>.05	4.79 ± 0.53	4.82 ± 0.45	>.05
White blood cell (*10^9^/L)	3.5–9.7	5.92 ± 1.27	5.76 ± 1.67	>.05	5.99 ± 1.39	6.20 ± 1.51	>.05
Hemoglobin (g/L)	110–160	145.0 ± 10.3	138.8 ± 11.0	>.05	140.9 ± 22.0	139.0 ± 10.1	>.05
Platelet (*10^9^/L)	100–400	205.0 ± 53.1	202.4 ± 55.9	>.05	205.1 ± 46.5	209.6 ± 49.5	>.05

### 3.4 Effects on serum biochemical parameters

Tables 5 showed the serum biochemical parameters for total serum protein, serum albumin, aspartate aminotransferase (AST), alanine aminotransferase (ALT), serum glucose, blood urea nitrogen (BUN), uric acid, and creatinine levels in obese subjects.

**Table 5:** Effects of intervention with placebo and PVE on biochemical parameters in obese subjects (Data are shown as means ± standard deviation)

	Placebo (*n* = 58)		*p* Value	PVE (*n* = 56)		*p* Value
	Baseline	35 Days		Baseline	35 Days	
Total serum protein (g/L)	70.1 ± 3.4	71.2 ± 3.6	>.05	71.1 ± 3.9	71.1 ± 3.7	>.05
Serum albumin (g/L)	43.8 ± 2.6	44.7 ± 2.8	>.05	44.6 ± 2.3	44.5 ± 2.2	>.05
Aspartate aminotransferase (U/L)	26.7 ± 9.9	27.7 ± 8.8	>.05	27.5 ± 7.2	26.3 ± 6.4	>.05
Alanine aminotransferase (U/L)	24.3 ± 7.4	25.6 ± 5.8	>.05	24.9 ± 6.7	26.2 ± 5.7	>.05
Blood glucose (mmol/L)	4.98 ± 0.51	5.08 ± 0.50	>.05	4.99 ± 0.60	5.02 ± 0.58	>.05
Blood urea nitrogen (mmol/L)	5.26 ± 1.49	5.44 ± 1.14	>.05	5.28 ± 1.18	5.26 ± 1.29	>.05
Uric acid (μmol/L)	309.8 ± 81.2	307.9 ± 64.8	>.05	317.7 ± 65.5	312.6 ± 58.8	>.05
Creatinine (μmol/L)	77.1 ± 14.2	75.7 ± 13.4	>.05	80.7 ± 16.6	80.9 ± 15.8	>.05

Before and after the trial, AST and ALT as markers of live function did not change and remain in the respective reference ranges of normality. BUN, uric acid, and creatinine as markers of kidney function were not significantly fluctuated when compared to baseline after 35 days intervention. Uric acid and creatinine levels were higher in the active group than the control group, but did not reach the significant difference. All the biochemical parameters including serum glucose in the two groups were within the normal range, and no abnormal fluctuation was observed during the intervention (Table 5). There were no significant changes in these parameters between the two groups before and after the trial.

### 3.5 Effects on electrocardiogram, chest X‐ray, and abdominal ultrasound examination and exercise tolerance intervention

Before the trial, there were no abnormalities in the electrocardiogram, chest X‐ray, and abdominal ultrasonography of two groups. There was no significant change in exercise tolerance between the two groups before and after the trial, as shown in Table 6.

**Table 6:** Effects of intervention with placebo and PVE on exercise endurance in obese subjects (Data are shown as means ± standard deviation)

	Placebo (*n* = 58)		*p* value	PVE (*n* = 56)		*p* value
	Baseline	35 Days		Baseline	35 Days	
Heart rate (time/min)^a^	158.1 ± 5.1	158.4 ± 5.3	>.05	159.2 ± 5.7	150.6 ± 6.4	>.05
Maximum oxygen consumption (L/min)	1.85 ± 0.13	1.85 ± 0.13	>.05	1.83 ± 0.09	1.96 ± 0.15	>.05

a Subject's heart rate measured by the 5‐min power cycling.

### 3.6 Determination of weight loss parameters

#### 3.6.1 Body weight and body fat

The results are shown in Table 7, and it was found that the PVE group has a significant weight loss after 35 days intervention (*p* < .01). The average decreased amount of the subjects’ weight by the PVE group was 2.24 kg (82.87 ± 4.75 kg to 80.63 ± 5.02 kg, *p* < .01), compared with a 0.29 kg weight loss (83.81 ± 4.72 kg to 83.52 ± 4.70 kg, *p* > .05) in placebo group after 35 days. The body fat mass lost an average of 1.95 kg, and the placebo group decreased 0.5 kg compared to baseline. The percentage of body fat decreased from 32.23 ± 3.28% to 30.69 ± 3.71%, lost by an average of 1.53%. And there was also a significant reduction in the overweight percentage, an average of decreased by 3.66%, compared to 0.45% reduction in the placebo group. BMI decreased by 0.79 kg/m^2^ in the PVE group and 0.1 kg/m^2^ in the placebo group. After 35 consecutive days, the PVE group have a significant reduction in body weight, body fat mass, body fat percentage, overweight percentage, and BMI at the baseline and at the end of trial (*p* < .01), whereas there was no significant change in the placebo group (*p* > .05). As Table 7 illustrates, there was also a significance difference (*p* < .05) between placebo and PVE groups in body weight, body fat mass, overweight percentage, and BMI after 35 days.

**Table 7:** Effects of intervention with placebo and PVE on body weight, body fat mass, body fat, overweight, and body mass index (BMI) in obese subjects

	Placebo (*n* = 58)		D‐value	*p* value	PVE (*n* = 56)		D‐value	*P* value
	Baseline	35 Days			Baseline	35 Days		
Body weight (kg)	83.81 ± 4.72	83.52 ± 4.70	0.29 ± 0.30	>.05	82.87 ± 4.75	80.63 ± 5.02*	2.24 ± 1.27**	<.01
Body fat mass (kg)	26.18 ± 2.43	25.68 ± 2.40	0.50 ± 0.61	>.05	26.61 ± 2.13	24.65 ± 2.61*	1.95 ± 0.44**	<.01
Body fat (%)	31.32 ± 3.25	30.28 ± 3.24	0.49 ± 0.66	>.05	32.23 ± 3.28	30.69 ± 3.71	1.53 ± 1.68**	<.01
Overweight (%)	32.60 ± 5.00	32.14 ± 5.04	0.45 ± 0.48	>.05	33.49 ± 5.26	29.82 ± 4.62*	3.66 ± 2.04**	<.01
BMI (kg/m^2^)	28.87 ± 0.83	28.77 ± 0.84	0.10 ± 0.10	>.05	28.97 ± 0.90	28.17 ± 0.82*	0.79 ± 0.44**	<.01

Note

Data are shown as means ± standard deviation. Among various parameters, a comparison of the individual changes (35 day values—baseline) was made both within groups and between the placebo and PVE groups. *p* values correspond to the comparison of baseline and 35 days within each treatment.

* Significant difference between the placebo and PVE groups (*p *< .05).

** Indicates significant difference between the placebo and PVE groups (*p *< .01).

#### 3.6.2 Subcutaneous fat thickness and waist/hip measurement

The subcutaneous fat thickness and waist/hip circumference are shown in Table 8. Before the trial, waist circumference, hip circumference, and subcutaneous fat thickness at four points (triceps, subscapular, abdomen, and suprailiac) were measured and there was no significant difference between the two groups (*p* > .05). After 5 weeks intervention, all the subcutaneous fat thickness at the four measuring points of the extract group decreased: an average decrease of 0.56 mm in the right triceps, an average decrease of 0.42 mm in the subscapular, an average decrease of 1.44 mm in the 3 cm next to right umbilicus, and the average drop in the suprailiac was 1.51 mm. After the trial meal of 5 weeks, there was a significant difference in waist/hip circumference between two groups. The waist circumference of the intervention group was reduced from an initial 105.5 ± 5.4 cm to 102.8 ± 5.6 cm (*p* < .01), decreased by an average of 2.7 cm. Hip circumference was changed from 119.8 ± 7.0 to 117.5 ± 7.4 cm (*p* < .01), decreased by 2.3 cm. There was no significant change on waist/hip circumference during this period in the placebo group. In summary, after statistical analysis, the comparisons between the above six indicators in the intervention group before and after the intervention was statistically significant (*p* < .01), and the decrease in each index of the intervention group was also significantly greater than that of the control group (*p* < .05 or *p* < .01); after the intervention meal, the waist circumference of the extract group was significantly smaller than that of the control group (*p* < .05). The above results showed that the skinfold fat thickness were significantly reduced after intake the extracts.

**Table 8:** Effects of intervention with placebo and PVE on subcutaneous fat thickness in obese subejcts

	Placebo (*n* = 58)		D‐value	*p* value	PVE (*n* = 56)		D‐value	*p* value
	Baseline	35 Days			Baseline	35 Days		
Triceps (cm)	45.93 ± 6.92	45.75 ± 6.96	0.18 ± 0.64	>.05	47.19 ± 8.03	46.63 ± 7.78	0.56 ± 1.17*	<.01
Subscapular (cm)	45.59 ± 6.95	45.48 ± 7.27	0.11 ± 0.65	>.05	46.09 ± 6.63	45.67 ± 6.62	0.42 ± 0.89*	<.01
Umbilical (cm)	21.50 ± 6.22	21.11 ± 6.09	0.39 ± 0.62	>.05	22.68 ± 6.26	21.25 ± 6.12	1.44 ± 1.17**	<.01
Subscapular (cm)	28.29 ± 6.86	28.04 ± 6.73	0.25 ± 0.69	>.05	30.42 ± 6.46	28.91 ± 6.38	1.51 ± 1.02**	<.01
Waist circumference (cm)	105.6 ± 5.7	105.3 ± 5.7	0.32 ± 0.51	>.05	105.5 ± 5.4	102.8 ± 5.6*	2.79 ± 1.60**	<.01
Hip circumference (cm)	120.1 ± 7.6	119.6 ± 7.6	0.48 ± 0.74	>.05	119.8 ± 7.0	117.5 ± 7.4	2.38 ± 1.71**	<.01

Note

Data are shown as means ± standard deviation. Among various parameters, a comparison of the individual changes (35 day values—baseline) was made both within groups and between the placebo and PVE groups. *p* values correspond to the comparison of baseline and 35 days within each treatment.

* Means value significantly different between the placebo and PVE groups (*p *< .05).

** Means value significantly different between the placebo and PVE groups (*p *< .01).

## 4 DISCUSSION

In this study, 114 subjects with simple obesity who passed physical examination (58 cases in the control group: 28 males and 30 females; 56 in the PVE group: male in 29 cases and 27 females) were performed in human daily meal intervention for weight loss. The active group administrated 2,400 mg PVE before each daily meal for 35 consecutive days, and the control group received placebo. As a result, the weight of the subjects dropped by an average of 2.24 kg after intake of PVE, which has a greater effect on body weight than does the placebo (weight loss of 2.24 kg vs. 0.29 kg). The average weight of overweight decreased 3.66%. The BMI index decreased by an average of 0.79, the total body fat decreased by 1.95 kg, and the body fat decreased by 1.53% on average compared to baseline. The thickness of subcutaneous fat was significantly reduced at the four measurement points, and the decrease of waist circumference and hip circumference was also significant. The differences of the various indicators between the two groups were significant before and after the study period, indicating that the PVE has weight loss function.

At the beginning and the end of intervention period, the subjects' hemoglobin, red blood cell, white blood cell and platelet counts, total serum protein, serum albumin, AST, ALT, creatinine, BUN, uric acid, and serum glucose were all within the normal range. Routine urine test, routine stool test, electrocardiogram, chest X‐ray, and abdominal ultrasound were normal and no change in exercise tolerance before and after the intervention, indicating that white kidney bean extract had no adverse effects on the health of the obese subjects. No allergies or other adverse reactions were observed during the total trial period.

Our results clearly indicated that oral supplementation of PVE capsules in the dose of 2,400 mg per day for 35 consecutive days resulted in a significant reduction in body weight, BMI, fat mass, subcutaneous fat thickness, and waist/hip circumferences compared to subjects receiving placebo. PVE administration has no adverse effects. This result was in consistent with many previous published results demonstrated that consumption of common beans (*Phaseolus vulgaris* L.) associated with antiobesity activity. Daily intake of half a cup of vegetarian baked beans over 8 weeks has proven to be favorable for hypercholesterolemic adults by decreasing serum triglyceride (6%) and LDL (5%) (Winham & Hutchins, 2007). Other studies conducted with another PVE—Phase 2 alpha‐amylase inhibitor, indicated that administration of PVE reduces the rate of absorption of carbohydrates, thereby reducing the GI of foods, decreased body weight gain in overweight subjects (Barrett & Udani, 2011). PVE is safe and effective for weight loss in a 12‐week randomized, double‐blind, placebo‐controlled study and maintain their weight over 24 weeks, where the subjects’ energy intake was ad libitum (Grube, Chong, Chong, & Riede, 2014). A randomized, double‐blinded, placebo‐controlled investigation in which body weights of generally healthy, overweight human volunteers in China were examined before and after 30 and 60 days of oral treatment with placebo or a starch blocker produced from white beans. After 60 days, 51 subjects receiving PVE compared with a placebo group of 50 subjects had clinical and statistically significantly greater average reduction of body weight [−1.9 kg vs. −0.4 kg, (*p* < .001)] and waist circumference [−1.9 cm vs. −0.4 cm, (*p* < .001)], but no difference in the changes of average hip circumference (Wu et al., 2010). A 4‐week randomized, double‐blind, placebo‐controlled study of 25 healthy subjects consuming 1,000 mg of a proprietary fractioned white bean extract or an identical placebo twice a day before meals was conducted. Subjects ate the most carbohydrates experienced a significant reduction in both weight and waist size with the addition of the white bean extract compared to the placebo group (Udani & Singh, 2007). Although some clinic studies demonstrated that the PVE exerted beneficial effects in weight loss, other studies showed there is no significant effect in weight loss compared to placebo. Fifty obese adults (BMI 30–43) were screened to participate in a randomized, double‐blind, placebo‐controlled study evaluating the effects of treatment with Phase 2 versus placebo on weight loss for 8 weeks, Body fat percentage, waist and hip circumferences, energy level, hunger, appetite, HbA1c, and total cholesterol have no clinically or statistically significant differences were identified between the active and the placebo group. No adverse events during the study were attributed to the study medication (Udani et al., 2004).

Nowadays, the prevalence of obesity has increased rapidly worldwide. Meanwhile, the important role of diet in the prevention and treatment of obesity is widely acknowledged. Many data supported a positive correlation between excess refined carbohydrates intake and obesity, since carbohydrates are among the macronutrients that provide energy and can thus contribute to excess energy intake and subsequent weight gain (van Dam & Seidell, 2007). Before crossing the intestinal wall, all complex carbohydrates (i.e., starches), in most cases glucose, must be hydrolyzed to their monosaccharide units. There are several enzymes involved in this process: α‐amylase present in saliva and pancreatic juice converts complex carbohydrates into oligosaccharides. Then, various other glucosidase enzymes (maltase, lactase, etc.) present in the brush border of the small intestine convert these oligosaccharides to monosaccharides that can then be absorbed into the body. Glucose and other monosaccharides generated through this process are transported via the hepatic portal vein to the liver. Monosaccharides that are not immediately utilized for energy are stored for future energy needs as skeletal tissue and glycogen in the liver or as fat (triglycerides) in adipose tissue, liver, and plasma (Celleno et al., 2007). Carbohydrates that are resistant to digestion in the intestine enter the colon, where they are fermented by colonic bacteria to produce short‐chain fatty acids (such as acetic, propionic, and butyric acids), carbon dioxide, and methane. Since the possible relationship of carbohydrates intake with body fatness, there has been a shift toward a reduction in carbohydrates, particularly refined carbohydrates, as an approach to reduce weight and the incidence or related disease risk (Preuss, 2009).

Detail information revealed that the bioactive ingredient of white kidney beans is phaseolin, which is a 7S, oligomeric globulin containing three polypeptide subunits (α, β, and γ) and accounts for total protein in *Phaseolus vulgaris* grains. White kidney bean is rich in alpha‐amylase inhibitors and has three isoforms of alpha‐amylase inhibitor (namely alpha‐AI, alpha‐AI2, and alpha‐AIL). Alpha‐amylase inhibitors have N‐terminally glycosylated glycoproteins, which are synthesized as a thermostable glycoprotein on the endoplasmic reticulum, stored in the vacuole. The alpha‐AI isoform has antiamylase activity in humans that can effectively block starch decomposition. This enzyme is found in the embryonic axes and cotyledons in the seed and not in other organs of the plant. It is not active against plant alpha‐amylases and is therefore classified as an antifeedant or seed defense protein (Moreno et al., 1990). Use of the starch blockers from common beans to control obesity and diabetes was a research issue in the 1980s. Due to recent improved extraction methods such as supercritical carbon dioxide extraction, fractionation and heat treatment have kept α‐amylase inhibition activity and led to demonstrable efficacy of the starch blockers in human (Skop & Chokshi, 2006). The mechanism of the *Phaseolus vulgaris* α‐amylase inhibitor action shows that the inhibitor is effective in preventing starch digestion by completely blocking access to the active site of the enzyme (Obiro et al., 2008). The mechanism behind the weight loss relies on the reported alpha‐amylase‐inhibiting activity of the PVE has been reported (Gibbs & Alli, 1998; Santimone et al., 2004). In vitro experiments, PVE has been shown to inhibit the activity of α‐amylase and may help promote weight loss by interfering with the digestion of complex carbohydrates to simple, absorbable sugars, potentially reducing carbohydrate‐derived calories (Layer, Zinsmeister, & DiMagno, 1986). Meanwhile, the rapid absorption of carbohydrates was delayed, which would favorably influence the insulin system for to lesser fat accumulation (Radulian, Rusu, Dragomir, & Posea, 2009). A growing evidence proved that gut microbiota plays a crucial role in the management of obesity and diabetes (Baothman, Zamzami, Taher, Abubaker, & Abu‐Farha, 2016; Guida & Venema, 2015). Additionally, PVE might prevent diet‐induced obesity and its related metabolic disorders through a beneficial influence on the gut microbiota. An administration of PVE in high‐fat diet‐induced obesity and obesity‐associated metabolic syndromes mice increased the relative abundance of *Bifidobacterium*, *Lactobacillus,* and *Akkermansia* at the genus level. This change might be sufficient to reverse high‐fat diet metabolic syndromes, including obesity, hyperglycemia, insulin resistance, and hepatic steatosis, without a major modification in the relative proportion of Firmicutes to Bacteroidetes (Song et al., 2016).

## 5 CONCLUSION

In summary, after analysis of the various effective parameters, our results clearly revealed that intake of dietary 2,400 mg PVE capsules daily by obese human subjects is more effective at reducing body weight and body fat mass compared to daily intake of placebo in a short time period. In our experiments, no clinically significant changes and adverse events occurred or felt due to the active product during the trial period. PVE has the potential to induce weight loss and reduce fat mass caused by carbohydrates through its alpha‐amylase inhibiting activity. This work provides the detail information for the depth study and wide applications of PVE in the future work.

## CONFLICT OF INTEREST

There is no conflict of interest. All the authors declare no conflict of interest. The lead author affirms that this manuscript is an honest, accurate, and transparent account of the study being reported.

## ETHICAL APPROVAL

The study conforms to the Declaration of Helsinki Guidelines for human subjects and approved by the Clinic Research Ethics Committee of Guangxi Zhuang Autonomous Region for Disease Prevention and Control.
