# Corrigendum

**DOI:** 10.1002/fsn3.1912

**Published:** 2020-09-30

In the article by Wang et al. (2020) There is an error in the second paragraph of the Introduction section. The correction is given below (in bold).

The white kidney beans are one species of Phaseolus vulgaris L., also known as common beans, is originated **from Mexico and Argentina**. Around 23 million tons were produced in the world in 2012, and the largest consuming region is South America (Heredia‐Rodríguez, de la Garza‐Juárez, Vazquez‐Rodriguez, 2017).

## References

[fsn31912-bib-0001] Wang, S. , Chen, L. , Yang, H. , Gu, J. , Wang, J. , & Ren, F. (2020). Regular intake of white kidney beans extract (*Phaseolus* *vulgaris* L.) induces weight loss compared to placebo in obese human subjects. Food Sciences and Nutrition, 8, 1315–1324. 10.1002/fsn3.1299 PMC7063375 32180941

